# Supplementary material for: Interactions between age and sex in multiscale entropy and spectral power changes across the lifespan
Source: Imaging Neurosci (Camb). 2026 Oct 22;4:IMAG.a.1407. doi: 10.1162/IMAG.a.1407 (PMC13697247; doi:10.1162/IMAG.a.1407)
Supplement: Supplementary file 1 [file IMAG.a.1407_supp1.pdf]

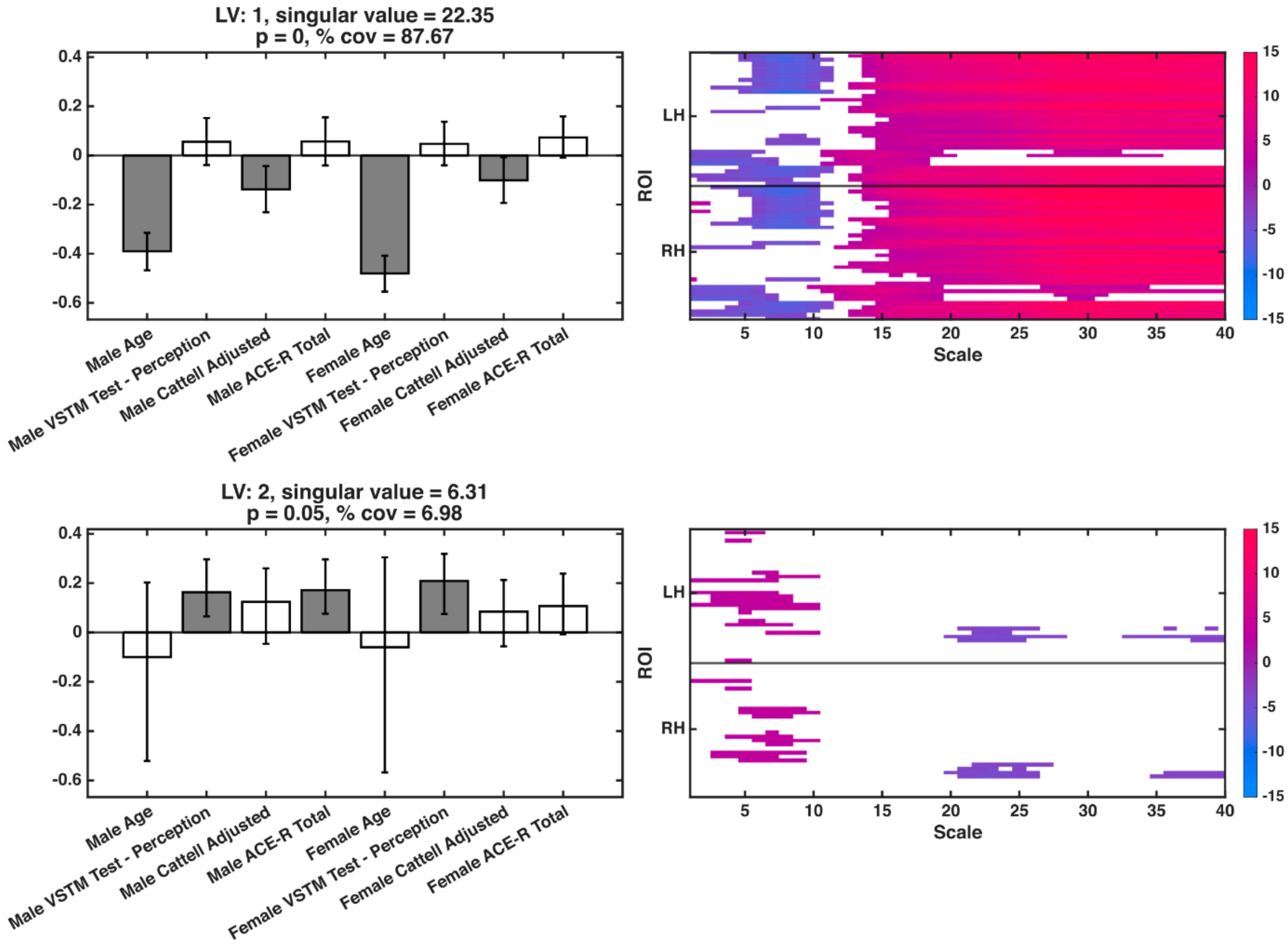

Supplementary Materials: Figure 1 - Results of the sex grouped MSE behavioural partial least squares analysis. Each significant LV, 1 and 2, are represented by two plots: 1) a bar plot of the correlation of each behaviour within each sex to the LV ± a 95% confidence interval; and 2) a colour plot of the bootstrap ratios for the MSE curves from each region interest (68 total, 34 in each hemisphere; RH = Right hemisphere, LH = Left hemisphere) indicating where correlations with behaviour, age, and sex were most reliable.
