# Supplementary material for: Interactions between age and sex in multiscale entropy and spectral power changes across the lifespan
Source: Imaging Neurosci (Camb). 2026 Oct 22;4:IMAG.a.1407. doi: 10.1162/IMAG.a.1407 (PMC13697247; doi:10.1162/IMAG.a.1407)
Supplement: Supplementary file 3 [file IMAG.a.1407_supp3.pdf]

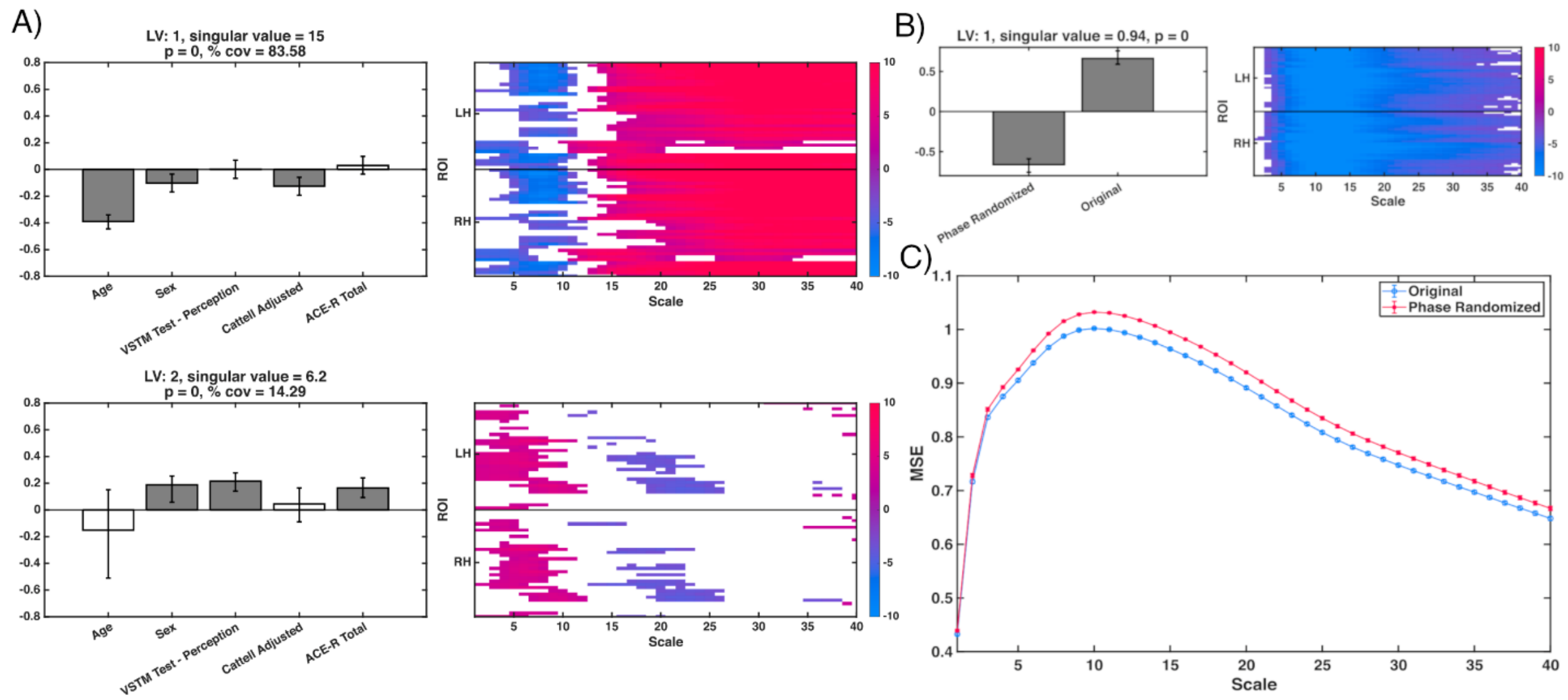

Supplementary Materials: Figure 3 - Results of the behavioural partial least squares (PLS) analysis of MSE curves derived from the phase-randomized data. A) Each significant LV, 1 and 2, are represented by two plots: 1) a bar plot of the correlation of each behaviour to the LV ± a 95% confidence interval; and 2) a colour plot of the bootstrap ratios for the phase-randomized MSE curves from each region of interest (68 total, 34 in each hemisphere; RH = Right hemisphere, LH = Left hemisphere) indicating where correlations with behaviour, age, and sex were most reliable. B) Results of a task PLS analysis comparing MSE curves derived from the original and phase randomized timeseries. The significant LV is represented by two plots: 1) a bar plot of the brain scores of each group ± a 95% confidence interval; and 2) a colour plot of the bootstrap ratios for the phase-randomized MSE curves from each region of interest indicating where the group effects were most reliable. C) Descriptive plots of the MSE curves derived from the original and phase-randomized timeseries [mean ± standard error].
